# The two-stage exploratory association between hypertension and physical examinations: a community-based biobank study

**DOI:** 10.3389/fcvm.2025.1357791

**Published:** 2025-10-27

**Authors:** Chih-Sheng Chen, Dai-Yin Chen, Ta-Chen Chen, Hsin-Yi Lo, Tse-Yen Yang

**Affiliations:** ^1^ Division of Chinese Medicine, Asia University Hospital, Taichung, Taiwan; ^2^ Department of Food Nutrition and Health Technology, Asia University, Taichung, Taiwan; ^3^ Department of Chinese Medicine, China Medical University Hospital, Taichung, Taiwan; ^4^ School of Post-Baccalaureate Chinese Medicine, China Medical University, Taichung, Taiwan; ^5^ Artificial Intelligence and Robotics Innovation Center (AIRIC), China Medical University Hospital, Taichung, Taiwan; ^6^ Graduate School of Pharmaceutical Sciences, Nihon Pharmaceutical University, Tokyo, Japan; ^7^ Master Program of Digital Health Innovation, College of Humanities and Sciences, China Medical University, Taichung, Taiwan; ^8^ Graduate Institute of Chinese Medicine, China Medical University, Taichung, Taiwan; ^9^ Molecular and Genomic Epidemiology Center & Department of Medical Research, China Medical University Hospital, Taichung, Taiwan; ^10^ Department of Medical Laboratory Science and Biotechnology, College of Medical and Health Science, Asia University, Taichung, Taiwan

**Keywords:** community-based biobank study, hypertension, exploratory study, clinical examinations, neural network

## Abstract

**Introduction:**

Hypertension is one of the most critical public health problems in developing countries and a leading cause of mortality and disability. Relevant extrapolations show that the global adult population with high blood pressure will increase significantly. This study utilizes partial data from the Taiwan Biobank to explore the association between clinical examinations and hypertension-related morbidities.

**Methods:**

Data for this study were sourced from the Taiwan Biobank, which has collected health information since 2012 and serves as a partial source for scientific research into lifestyle and health trends of cardiovascular-related diseases in the general population. This study focused on distinguishing the correlation between blood pressure changes and stability in various age groups and exploring the relationship between environmental exposure factors and health behaviors through stratified analysis.

**Results:**

The comorbidities identified as significant risk factors for hypertension include hyperlipidemia [odds ratio (OR), 4.0504] and diabetes (OR, 2.1871). Clinical biochemistry examinations also indicated classifiers for hypertension, such as age, heart rate, triglycerides, high-density lipoprotein cholesterol, hyperlipidemia, and diabetes, which were represented as explanatory indicators [the area under the receiver operating characteristic curve (AUROC), 0.8769]. These findings underscore the potential of clinical examinations to predict and prevent hypertension.

**Conclusion:**

This study suggested the possibility of developing a risk assessment tool based on these classifiers and investigated the generalizability of these findings using biobank resources. The findings could aid in informing clinical decision-making, enhancing digital health education, and reducing the burden of hypertension.

## 1 Introduction

Hypertension (HT) remains a critical public health challenge in developing countries and continues to be a leading cause of mortality and disability globally (1). Relevant studies and inferences indicate that, by 2025, the global adult population suffering from hypertension is expected to increase to a quarter (approximately25%) (2), with prevalence potentially rising to 29% (3). Given its high prevalence and association with severe cardiovascular disorders such as stroke and increased mortality in individuals with diabetes (4, 5), effective management of hypertension is paramount, even if it may also cause many other cardiovascular-related disorders (5). Prior research has emphasized the importance of comprehensive health management, including blood pressure control and heart rate monitoring, in reducing mortality among individuals with hypertension (6). The mortality rate of individuals with better blood pressure control or a slower heart rate is lower than that of those without comprehensive health management (6). Without well-controlled hypertension, it might lead to elevated mortality and complications in adults with diabetes (4). In addition, well-managed hypertension (4), daily routine exercise (7), and a balanced diet (8–10) are also possible factors affecting blood pressure.

Community-based biobank health data serve as the source of data science research to explore and summarize the lifestyle and health trends of cardiovascular-related diseases among the healthy and sub-healthy groups in the general population (11–13). They can be used to distinguish the correlation between blood pressure control and maintaining numerical stability and conduct stratified analysis to explore the correlation between environmental exposure and healthy behavior (14–16). The present study focuses on the clinical examination and exploration of the association with hypertension, aiming to investigate the explanatory potential of health surveillance data in the biobank dataset. However, despite the well-documented impact of hypertension and the influence of lifestyle factors, a detailed understanding of the specific associations between routine clinical examinations and the subsequent development of hypertension-related morbidities within the biobank's baseline survey remains limited. Therefore, this study aims to leverage the rich dataset of the Taiwan Biobank to explore these associations, potentially identifying key clinical indicators that could inform more targeted prevention and management strategies for hypertension in this population.

## 2 Materials and methods

### 2.1 Data sources and participatory

A sub-dataset of 1,998 participants was drawn from the Taiwan Biobank dataset (17), which collects Taiwanese individuals aged 30–70 years old without cancer incidents. These volunteers were recruited from the various Taiwan Biobank recruitment sites. The present sub-dataset involved 1,998 participants in a partial Taiwan Biobank, and individual health questionnaires were collected from the Taiwan Biobank station. Detailed health information was available from the Taiwan Biobank from 2012 to the present.

These authors assert compliance with the Helsinki Declaration of 1964, as revised in 2013, and the Taipei Declaration of 2016 on ethical considerations regarding biobanks, ensuring that all essential ethical aspects of this work also comply with relevant ethical guidelines and regulations on human subjects' research and human biobank management in Taiwan. Moreover, informed consent was obtained from all the participants included in the Taiwan Biobank. The study was approved by the institutional review board of China Medical University Hospital (CRREC-105-068-CR8) and the Taiwan Biobank Ethics Governance Committee (TWBR10512-01).

### 2.2 Essential characteristics and outcomes analysis

The questionnaires generated primary characteristic data, including self-reported diseases and subsequent clinical examinations, to represent individual health status at baseline inclusion. The preliminary data were comprehensively obtained from a cross-sectional, community-based study design in the Taiwan Biobank. Thus, most morbidities, such as gout, coronary artery disease, hyperlipidemia, hypertension, and diabetes, were self-reported by the general population in Taiwan. The outcomes were based on self-reported hypertension, with participants indicating no hypertension or missing data classified as non-hypertensive in the present dataset. Participants with missing data for the variables included in the analyses were regrouped through listwise combination as non-patients. The univariate analysis was applied to calculate the essential characteristics for estimating mean or proportion difference using the Fit Y by X module of JMP Academic Suite Version 18.1.2 (JMP Statistical Discovery LLC., Cary, NC, USA). The clinical examination indicators demonstrated different patterns between self-reported hypertension and non-hypertension, which inclued (1) general practice data (height, weight, fat rate, and waistline); (2) hematological variables [red blood cell (RBC) count, white blood cell (WBC) count, platelet count, hemoglobin (Hb), and hematocrit (Hct)]; (3) biochemical variables [glycated hemoglobin (HbA1c), glucose AC (ante cibum, means before meals), total cholesterol, high-density lipoprotein cholesterol (HDL-C), low-density lipoprotein cholesterol (LDL-C), and triglyceride levels]; (4) biochemical liver function [total bilirubin, albumin, SGOT (serum aspartate aminotransferase, also named as AST), and SGPT (serum glutamate pyruvate transaminase, also named as ALT) levels]; and (5) kidney function [blood urea nitrogen (BUN), creatinine, and uric acid levels]. Further clinical examination variables were collected from the Taiwan Biobank station and examined at the core lab of Chang-Gung Memorial Hospital, Linkou Branch, to avoid systemic errors. A control chart graph builder was used to monitor the main changes and assess age-related biosignatures in the biobank, utilizing general practice medical data from various age groups.

### 2.3 Statistical analysis

The JMP Academic Suite Annual Professor/Researcher license was used in all statistical analyses, divided into SAS JMP and JMP Pro Version 18.1.2 (JMP Statistical Discovery LLC., Cary, NC, USA). Categorical and continuous variables were analyzed separately using the chi-square test and *t*-test to determine significance, with a *p*-value of <0.05 as the threshold. The area under the receiver operating characteristic (AUROC) estimation was used in the predictive modeling module to estimate the probability of predictors. Moreover, the control chart builder was applied to test the clinical examination indicator and observe indigenes in the population to assess the mean differences among various age periods, as life changes are reflected in general practice information.

## 3 Results

### 3.1 The primary characteristics of two thousand volunteers

A total of 2,000 people were included in the analysis, with two dropouts. Among the self-reported hypertension groups, the average age of the group without hypertension (HT, *n* = 1,771) was 47.40 years (SD, 10.58), and the average age of the group with hypertension (non-HT, *n* = 227) was 56.21 years (SD, 8.62). Among the gender variants, women accounted for 52% of the non-hypertensive group, whereas men accounted for 69% of the hypertensive group. In the physiological examination, systolic blood pressure (HT vs. non-HT, 135.10 ± 16.52 vs. 113.16 ± 15.88, *p* < 0.0001), diastolic blood pressure (HT vs. non-HT, 80.88 ± 10.90 vs. 70.98 ± 10.91, *p* < 0.0001), body waistline (HT vs. non-HT, 89.29 ± 8.95 vs. 83.50 ± 9.49, *p* < 0.0001), body mass index (BMI; HT vs. non-HT, 25.98 ± 3.27 vs. 24.18 ± 3.51, *p* < 0.0001), urea nitrogen (HT vs. non-HT, 14.70 ± 3.72 vs. 12.91 ± 3.48, *p* < 0.0001), creatinine (HT vs. non-HT, 0.84 ± 0.21 vs. 0.74 ± 0.20, *p* < 0.0001), uric acid (HT vs. non-HT, 6.12 ± 1.45 vs. 5.54 ± 1.48, *p* < 0.0001), fasting blood glucose (HT vs. non-HT, 105.89 ± 29.16 vs. 94.42 ± 17.00, *p* < 0.0001), glycated hemoglobin (HT vs. non-HT, 6.13 ± 1.12 vs. 5.70 ± 0.68, *p* < 0.0001), high-density cholesterol (HT vs. non-HT, 49.30 ± 10.98 vs. 54.61 ± 13.62, *p* < 0.0001), heartbeat speed (HT vs. non-HT, 68.81 ± 9.16 vs. 70.23 ± 9.05, *p* = 0.0268), and triglycerides (TG, HT vs. non-HT, 133.03 ± 79.32 vs. 116.37 ± 88.99, *p* = 0.0097) were statistically significantly different with or without hypertension (*p* < 0.05). However, there were no statistically significant differences between body fat rate (HT vs. non-HT, 27.61 ± 7.20 vs. 27.37 ± 7.14, *p* = 0.6397), total cholesterol (T_CHO, HT vs. non-HT, 192.50 ± 32.59 vs. 195.80 ± 36.45, *p* = 0.1943), low-density cholesterol (LDL_C, HT vs. non-HT, 121.99 ± 28.66 vs. 123.09 ± 33.35, *p* = 0.6351), and hypertension. In the history of related diseases, in the hypertension group, 96% reported no history of gout, 96% no history of hyperlipidemia, and 97% no history of diabetes.

**Figure 1:**
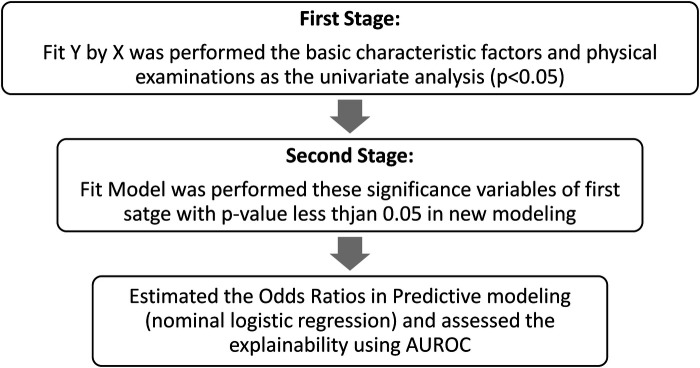
The workflow of two-stage exploration. The comprehensive analysis workflow followed the first stage (univariate analysis), the second stage (multivariate analysis), and predictive modeling (AUROC estimation) for clarifying the relative risk (odds ratio) for hypertension in a biobank study.

In the hypertensive group, individuals without a history of gout (89%), hyperlipidemia (78%), or diabetes (85%) were more prevalent than those with these conditions. A history of alcoholism was reported in 99% of individuals, compared with <1% with no such history. Gout, hyperlipidemia, and diabetes mellitus (*p* < 0.0001) showed a statistically significant association with hypertension, while alcoholism did not. The characteristics of self-report hypertension are shown in Table 1. The exploration workflow followed a two-stage approach (Figure 1), with univariate analysis as the first stage, the multivariate analysis as the second, and predictive modeling using AUROC estimation to assess the relative risk and odds ratio (OR) for hypertension in the subsequent biobank datasets.

**Table 1:** Basic characteristic properties of participants with self-reported hypertension.

Dependent variables	Without self-reported hypertension^a^^,^^b^ (*n* = 1,771)	With self-reported hypertension^a^^,^^b^ (*n* = 227)	*p*-Value^c^^,^^d^
Basic variables			
Age	47.40 (10.58)	56.21 (8.62)	<0.0001
Sex			<0.0001
Female	52%	31%	
Male	48%	69%	
Physiological examinations			
Systolic pressure	113.16 (15.88)	135.10 (16.52)	<0.0001
Diastolic pressure	70.98 (10.91)	80.88 (10.90)	<0.0001
Body_Waistline	83.50 (9.49)	89.29 (8.95)	<0.0001
BMI	24.18 (3.51)	25.98 (3.27)	<0.0001
BUN	12.91 (3.48)	14.70 (3.72)	<0.0001
Creatinine	0.74 (0.20)	0.84 (0.21)	<0.0001
Uric_Acid	5.54 (1.48)	6.12 (1.45)	<0.0001
Body_Fat_Rate	27.37 (7.14)	27.61 (7.20)	0.6397
Heartbeat_Speed (beats per half minute)	70.23 (9.05)	68.81 (9.16)	0.0268
Fasting_Glucose	94.42 (17.00)	105.89 (29.16)	<0.0001
HbA1c	5.70 (0.68)	6.13 (1.12)	<0.0001
T_CHO	195.80 (36.45)	192.50 (32.59)	0.1943
TG	116.37 (88.99)	133.03 (79.32)	0.0097
HDL_C	54.61 (13.62)	49.30 (10.98)	<0.0001
LDL_C	123.09 (33.35)	121.99 (28.66)	0.6351
Morbidities			
Gout			<0.0001
Yes	77	26	
No	1,694	201	
Hyperlipidemia			<0.0001
Yes	67	50	
No	1,704	177	
Diabetes			<0.0001
Yes	45	34	
No	1,726	193	
Alcoholism			1.0000
Yes	2	0	
No	1,769	227	

^a^ These continuous variables were shown by mean (standard deviation).

^b^ These category variables were present in different proportions or numbers.

^c^ These *p*-values of continuous variables were calculated by logistic regression fitting.

^d^ These *p*-values of category variables were estimated using a two-tailed Fisher's exact test.

### 3.2 Logistic regression analysis

Logistic regression was used to calculate and analyze factors correlated with the presence or absence of hypertension. Significant correlations were found for systolic blood pressure (*p* < 0.0001), hyperlipidemia (*p* < 0.0001), age (*p* < 0.0001), heart rate (*p* = 0.0003), high-density cholesterol (*p* = 0.0153), diabetes mellitus (*p* = 0.0275), and triglyceride level (*p* = 0.0396). Diastolic blood pressure (*p* = 0.1244), creatinine (*p* = 0.0168), fasting blood glucose (*p* = 0.3182), BMI (*p* = 0.3694), uric acid (*p* = 0.4174), gout (*p* = 0.4618), urea nitrogen (*p* = 0.5671), sex (*p* = 0.6393), waist circumference (*p* = 0.8366), and glycated hemoglobin (*p* = 0.8797) were not associated with hypertension. The logistic regression results for self-reported hypertension are shown in Table 2.

**Table 2:** Logistic regression for self-reported hypertension.

Dependent variables	Wald chi-square	Prob > chi-square
Systolic_Pressure (mmHg)	56.03	<0.0001**
Hyperlipidemia	31.70	<0.0001**
Age (years)	26.29	<0.0001**
Heartbeat_Speed (beats per half minute)	13.11	0.0003**
HDL_C	5.89	0.0153*
Diabetes (mmHg)	4.86	0.0275*
TG	4.24	0.0396*
Diastolic_Pressure (mmHg)	2.36	0.1244
Creatinine	1.90	0.1680
Fasting_Glucose	1.00	0.3182
BMI	0.81	0.3694
Uric_Acid	0.66	0.4174
Gout	0.54	0.4618
BUN	0.33	0.5671
Sex (male = 1; female = 2)	0.22	0.6393
Body_Waistline (cm)	0.04	0.8366
HbA1c	0.02	0.8797

*p < 0.05; **p < 0.001.

HDL_C, high-density lipoprotein cholesterol; TG, triglyceride; BMI, body mass index; BUN, blood urea nitrogen; HbA1c, glycohemoglobin.

### 3.3 Odds of self-reported hypertension between related risk factors and hypertension

In multivariate analysis, age [OR, 1.06; 95% confidence interval (CI), 1.03–1.08; *p* < 0.0001], systolic blood pressure (OR, 1.05; 95% CI, 1.04–1.06; *p* < 0.0001), heart rate (OR, 0.97; 95% CI, 0.95–0.98; *p* = 0.0002), triglycerides (OR, 1.0; 95% CI, 0.99–1.0; *p* < 0.0256), high-density cholesterol (OR, 0.98; 95% CI, 0.0. 96–1.0; *p* < 0.0131), hyperlipidemia (OR, 4.05; 95% CI, 2.49–6.6; *p* < 0.0001), diabetes mellitus (OR, 2.9; 95% CI, 1.09–4.39; *p* < 0.0275) were significantly associated with hypertension in all study participants (as shown in Table 3). This study showed that age, systolic blood pressure, heart rate, triglycerides, high-density cholesterol, hyperlipidemia, and diabetes were associated with hypertension. Further understanding the potential risk factors that contribute to hypertension will help reduce the incidence of hypertension in the Taiwanese population.

**Table 3:** The relative risk for self-reported hypertension using odds ratio estimation.

Dependent variables^a^	Odds ratio	Lower 95%	Upper 95%	*p*-Value^b^
Unit odds ratio				
Age	1.0552	1.0338	1.0771	<0.0001**
Systolic_Pressure	1.0539	1.0395	1.0685	<0.0001**
Diastolic_Pressure	1.0167	0.9954	1.0384	0.1232
Body_Waistline	1.0035	0.9704	1.0378	0.8367
BMI	1.0421	0.9523	1.1403	0.3703
BUN	0.9849	0.9348	1.0376	0.5658
Creatinine	2.3251	0.7007	7.7160	0.1592
Uric_Acid	1.0606	0.9200	1.2228	0.4172
Heartbeat_Speed	0.9655	0.9473	0.9840	0.0002**
Fasting_Glucose	1.0064	0.9939	1.0190	0.3157
HbA1c	1.0270	0.7273	1.4502	0.8798
TG	0.9973	0.9947	0.9999	0.0256*
HDL_C	0.9799	0.9640	0.9961	0.0131*
Odds ratio				
Sex	1.1314	0.6752	1.8960	0.6393
Gout	1.2514	0.6886	2.2741	0.4618
Hyperlipidemia	4.0504	2.4890	6.5912	<0.0001**
Diabetes	2.1871	1.0904	4.3868	0.0275*

* *p* < 0.05, **p < 0.001.

^a^ The continuous variables used the unit odds ratio to estimate the relative risk for hypertension; the odds ratio was calculated for the categorical variables as relative risk.

^b^ These *p*-values were used in the likelihood ratio tests to estimate the significance as *p* < 0.05.

### 3.4 Analysis of neural network patterns

**Figure 2:**
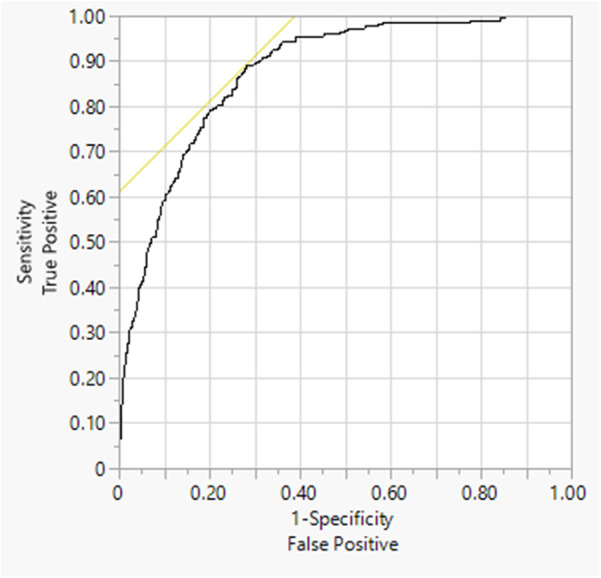
The AUROC for self-reported hypertension. The AUC (0.8769) was estimated by nominal logistic regression: age, systolic pressure, heartbeat speed, triglyceride, HDL-cholesterol, self-reported hyperlipidemia, and self-reported diabetes.

Using the database dataset, the possibility of preset pattern construction is provided by analyzing neural network patterns between questionnaire information and disease history. The correlation analysis and evaluation of physiological indexes and hypertension revealed that age, heart rate, triglycerides, high-density cholesterol, hyperlipidemia, and diabetes had a reasonable AUC (0.8769), indicating a notable specificity and predictive sensitivity (Figure 2). Nominal logistic regression (NLR) is a statistical method used for multi-class classification where the output categories have no inherent order. At the same time, a neural network is a computational model inspired by the human brain, capable of learning complex patterns and often used for classification and regression tasks.

## 4 Conclusion and discussion

The present study has several limitations and recommendations from different perspectives. First, the data collected from the Taiwan Biobank requires participants to be 30–70 years old and predominantly community health participants (18–20). Individuals under the age of 30 and over 70 years old were excluded. Therefore, the relative risk factors of the population with mild hypertension could not be incorporated into the analysis.

Biobanks are invaluable resources for disease-related studies but come with certain limitations, such as breakdown of challenges. (1) Samples in biorepositories can degrade over time due to factors such as temperature fluctuations or improper storage, affecting the accuracy of downstream analyses. Ensuring consistent and high-quality sample processing and storage is crucial but challenging. The participants' specimens process strategy was limited to the process interval within 70 min before frozen storage and cold shipment within 24–72 h. Thus, the specimens stored would be limited to the least interference from temperature disturbance. (2) Biobanks may collect data using different protocols, leading to data quality and format inconsistencies, so harmonizing data across different biobanks is essential for large-scale studies. However, the Taiwan Biobank was centrally managed and had all the stations for participant enrollment with standardized questionnaires, sample collection protocols, etc., which were covered by over 100 standard protocols. (3) The depth and accuracy of phenotypic data (information about an individual's observable characteristics) can vary significantly. Incomplete or inaccurate phenotypic data can limit the ability to identify associations between genetic factors and disease. (4) The ethical and legal considerations represent privacy and confidentiality, which means that protecting the privacy of biobank participants is paramount, and balancing data sharing with the intent to preserve individual privacy is another challenging issue. Further data sharing and access to establishing transparent and equitable data sharing policies are crucial for promoting collaborative research, simplifying data accessibility while protecting intellectual property, and ensuring responsible data leadership. However, this can be problematic. Under the centralized management of the ethical governance committee and the information security protocol mentioned, the informative management was approaching the trusted research environments. (5) Biobank participants commonly consider the lack of generalizability in representing the general population. However, the Taiwan Biobank enrollment covered 33 Taiwanese stations for participants' enrollment, which should solve the generalizability problem. Thus, the Taiwan Biobank could play a role as an established enrollment and management of biobank data and biorepository resources for innovative exploration of digital health studies. Our complete case analysis, while straightforward, might have introduced selection bias if the missing data were not entirely at random. This could potentially affect the generalizability of our findings; thus, these participants' data collection played a critical role in the edge relations, as evidenced by the referencing distribution in the Taiwan population.

In the disease status section, when asked whether a doctor has ever diagnosed hypertension, only the participant responds by himself and is not supported by the health insurance database data, which is prone to data deviation. Taiwan will enter a super-aging society by 2025, and the national health policy is also geared toward advanced deployment before the onset of diseases.

It is hoped that in the future, the data collection from Taiwan's human biological database can be combined with the data of the health insurance database, and the concept of data cloud can be integrated with cloud technology to increase the correctness of data and help the research and discussion of digital health data (21).

Hypertension is a common chronic disease because of lifestyle changes (5, 22), diet (8, 22, 23), and habits (24). In the coming decade, one in four people will have hypertension problems worldwide. Age (25, 26), sex (27), smoking, alcoholism, and lack of exercise (28) are risk factors for prehypertension, and prehypertension has higher glycemic values, triglycerides, and BMI. Therefore, by analyzing biobank data, we can understand the difference between hypertensive patients' physiological indicators and health status, and whether they have hypertension. The unit odds ratio represents the change in odds for a one-unit increase in the predictor variable. In contrast, the odds ratio can refer to comparing odds between any two categories or levels of a predictor variable. An odds ratio is a statistical measure used in research to determine the strength of association between two events. For example, in medical research, it might be used to compare the odds of an inevitable outcome occurring in a treatment group vs. a control group. A unit odds ratio focuses on a one-unit change in a continuous predictor variable, such as age and other continuous variables of clinical examinations. A general odds ratio might compare the odds of an outcome between two groups, such as self-reported hypertension (case) vs. non-hypertension (control). It provides a broader comparison without the specificity of a one-unit change. Both measures are crucial for understanding relationships in data, but they apply to different contexts and interpretations.

The case data from in-hospital health examinations are utilized to identify factors influencing hypertension. Relevant units are provided to prompt patients to return, receive prompt medical education, and undergo preventive treatment for high-risk patients—the concept of future medical institutions for public health consideration and education aimed at facilitating hypertension prevention.

In 2012, the Taiwan Biobank database started to recruit community health participants to join, including a total of approximately 184,577 community health participants (https://www.biobank.org.tw/statistics.php) aged 30–70 without cancer, of which approximately 45,439 participated in tracking the recipients. Taiwan Biobank management includes de-identification and information security management. The database contains detailed questionnaires covering five major topics: (1) general information (health status, environmental questionnaire, dietary behavior, family history of the disease, economic situation, CM-CQ, etc.); (2) physiological indicators (height, weight, body fat, waist-hip ratio, blood pressure, bone density, and lung function); and (3) routine hematology (RBC, WBC, platelets, heme, Hct, HbA1c), clinical biochemistry (fasting blood glucose, total cholesterol, triglycerides, HDL, LDL), liver function index [total bilirubin, albumin, AST, ALT, γ-GT (gamma-glutamyl transpeptidase), AFP (alpha-fetoprotein)] and renal function indicators (BUN, creatinine, uric acid, microalbumin), and HBV (hepatitis B virus)/HCV (hepatitis C virus) serology (anti-HCV antibody, HBsAg antigen, anti-HBs antibody, anti-HBe antibody, HBeAg antigen) and value-added genetic information (whole genome chip data, whole gene sequencing data, DNA methylation chip data, HLA (human leukocyte antigen) genotyping, and blood metabolite mapping, etc.). The Taiwan Biobank was a national database and specimen collection program that utilized the database to facilitate the construction of preset models through neural network analysis, linking questionnaire information to disease history. NLR, also known as multinomial logistic regression, is a statistical technique used for modeling and predicting outcomes where the dependent variable is nominal, meaning it consists of multiple categories that do not have a natural order or ranking, which extends beyond the binary logistic regression, which is limited to two possible outcomes, by allowing more than two categories. NLR estimates the probability of each category occurring based on the predictor variables. The model uses a set of coefficients to weigh each predictor, translating into each specific outcome's likelihood. The mathematical foundation of NLR involves the softmax function, a generalization of the logistic function to multiple classes. This ensures that the probabilities of all outcomes sum to one. One significant advantage of NLR is its interpretability. The coefficients derived from the model provide insights into how each predictor variable influences the probability of each outcome.

Neural networks require enormous amounts of data and computational resources to train effectively; thus, they are often seen as “black boxes” because their internal workings are less interpretable than simpler models such as logistic regression. Understanding why a neural network makes a particular decision can be challenging, which can be a drawback in applications where transparency and interpretability are essential. Therefore, NLR and neural networks are powerful tools for classification tasks, each with unique strengths and applications. NLR is valuable for its interpretability and simplicity, making it suitable for problems with a moderate number of categories and predictor variables. Neural networks are well-suited for handling more intricate and high-dimensional data, as they possess the ability to learn complex patterns and efficiently process large datasets. However, NLR should provide more interpretability and predictive power in statistical analysis.

In the past, research was usually designed in the hospital field, different from research using national biological databases. There are differences in the applicability of the general public, and the application of supervised analysis tools inspires us to use genomic analysis in modern and scientific Chinese medicine research, in addition to CM-CQ as an estimator for assessing disease-related risks, which will also contribute to the development of novel biotechnology pharmaceutical technologies in Chinese medicine (16, 28).

The national biobank database in Taiwan enables researchers to share de-identified digital health data. To access the database, researchers must apply to the data management authority and obtain approval before receiving the released data (18). Such an application may take more than several months, resulting in many researchers needing help to conduct data research smoothly due to the difficulty and complexity of the application. After establishing the relevant regulations of the Human Research Law, the national biobank has set up an ethical governance committee to manage and accelerate the application and review procedures, so that the time for researchers to obtain data has gradually decreased from more than six months to several months. Researchers can use de-linked or de-identified data on all participants to explore digital health data and examine the research hypothesis as new study resources.

In the context of receiver operating characteristic (ROC) curves, AUC is a measure of the model's ability to distinguish between classes. Our model outperforms random guessing, achieving a classification accuracy of 0.8769. It predicts both positives and negatives accurately, indicating that it surpasses a random model in predictive capability. However, it may still require refinement in sensitivity to detect failures more effectively. Our results revealed a connection between the classifier and hypertension through multiple clinical examinations and comorbidities, leading to implications for clinical practice and complex clinical observations. Therefore, examinations should carefully consider these indicators. The unmet solution should focus on what arises that warrants further clinical investigation or participant follow-up, which should be a significant issue for further approaches.

A significant limitation is our reliance on self-reported diagnoses of hypertension and related morbidities. This methodology inherently excludes individuals who are unaware of their condition, have not yet been diagnosed, or choose not to report their disease. Consequently, the prevalence of these conditions is underestimated in our cohort, which could potentially weaken the observed associations and limit the applicability of our results to the general population, which includes undiagnosed cases.

In the past, medical diagnoses were based on the patient's discomfort complaints, reasonable examination, routine clinical examination, examination results, and the judgment of the doctor with clinical experience. With the development of medical information technology, “precision medicine” is the current trend. In addition to using traditional methods for differential diagnosis, biomedical tests (28, 29) such as genes and proteins are added to compare and analyze personal information, such as gender, height, weight, past medical history, and family history. Biobank databases will be a companion to find out the most suitable treatments and prescription candidates for patients in the future. The difference between precision medicine and precision health was attributed to the different phenotypes of illness progression. Precision medicine is to explore the correlation between diseases through effective medical and scientific research, accurately understand the occurrence and progression of conditions, and try to make secondary prevention and limit disability. In the future, the concept of precision medicine can be transformed to link novel potential disease risk factors, integrate the situation of the environment and lifestyle, conduct a risk assessment, promote health status, shift health maintenance from treatment to prevention, and generate a higher quality of healthcare for the general population as we approach precision health. While the associations between factors such as BMI, cholesterol, and hypertension are well-documented, the principal contribution of this study is threefold. (1) It validates these findings within a large, contemporary Taiwanese population using biobank data, confirming their relevance in this specific demographic. (2) It demonstrates the utility of a two-stage exploratory approach in efficiently analyzing complex biobank datasets. (3) It provides up-to-date, population-specific estimates that are crucial for public health planning and clinical risk stratification in Taiwan.

## Data Availability

The original contributions presented in the study are included in the article/Supplementary Material; further inquiries can be directed to the corresponding author.
